# Major structural congenital anomalies and causal pathways in people with cerebral palsy

**DOI:** 10.1111/dmcn.16073

**Published:** 2024-09-05

**Authors:** Susan M. Reid, Gina L. Hinwood, Angela T. Guzys, Rod W. Hunt, Dinah S. Reddihough

**Affiliations:** ^1^ Neurodisability and Rehabilitation Murdoch Children's Research Institute Parkville Victoria Australia; ^2^ Department of Paediatrics University of Melbourne Parkville Victoria Australia; ^3^ Neurodevelopment and Disability The Royal Children's Hospital Parkville Victoria Australia; ^4^ Victorian Paediatric Rehabilitation Service Monash Children's Hospital Clayton Victoria Australia; ^5^ Department of Perinatal Medicine The Mercy Hospital for Women Heidelberg Victoria Australia; ^6^ Department of Paediatrics School of Clinical Sciences, Monash University Clayton Victoria Australia; ^7^ Monash Newborn Monash Children's Hospital Clayton Victoria Australia; ^8^ Cerebral Palsy Alliance University of Sydney Sydney New South Wales Australia

## Abstract

**Aim:**

To determine the proportion of persons with cerebral palsy (CP) with major congenital anomalies, factors associated with the presence of anomalies, body systems involved, potential contribution to CP aetiology, and causal pathway subgroups implicated.

**Method:**

This population‐based, observational study involved a cohort of 2238 persons born in one Australian state between 1999 and 2017. Major congenital anomalies were classified as affecting cerebral, cardiac, or other body systems, with further categorization as single or multisystem. We determined the potential for anomalies to contribute to the development of CP across causal pathway subgroups that were broadly categorized as developmental or involving destructive brain insults.

**Results:**

Of persons with CP, 23% had major congenital anomalies and 17% of the cohort had anomalies that potentially contributed to the development of CP. Consistent with higher odds of parental consanguinity, maternal grand multiparity, and dysmorphic features in the group with anomalies, 82% of pathogenic anomalies, present in 14% of the cohort, were cerebral and involved developmental causal pathways. Only 3% (predominantly severe cardiac anomalies) were related to destructive brain insults.

**Interpretation:**

The study provides context for the impact on rates of CP of preventive measures or other changes in incidence or management of congenital anomalies.

## Graphical abstract

Congenital anomalies are more common in people with cerebral palsy (CP) compared to the non‐CP population. In our population‐based study, 23.4% of persons with CP had a congenital anomaly (CA) but 7% of the cohort had anomalies that were unlikely to be pathogenic. Cerebral anomalies and developmental causal pathways comprised 14% of CP whereas congenital anomalies and pathways involving destructive brain insults accounted for less than 3% of CP.

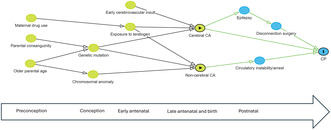

Plain language summary: https://onlinelibrary.wiley.com/doi/10.1111/dmcn.16148

This original article is commented by Damkjær on pages 282–283 of this issue.

**What this paper adds**

- Of persons with cerebral palsy (CP), 23.4% had a congenital anomaly but 7% of the cohort had anomalies that were unlikely to be pathogenic.
- Cerebral anomalies and developmental causal pathways comprised 14% of CP.
- Congenital anomalies and pathways involving destructive brain insults accounted for 3% of CP.

**Abbreviation:**
EUROCAT: European Surveillance of Congenital Anomalies

Cerebral palsy (CP) rarely has a sole cause; more often, a sequence or combination of antecedent factors contributes to the risk of brain pathology and CP along causal pathways.^1^ There has been recent interest in elucidating the role of congenital anomalies in causal pathways to CP.^2^ Congenital anomalies occur more frequently in CP cohorts compared to the wider birth population,^3^, ^4^, ^5^ particularly in children born near term.^4^, ^6^, ^7^ Based on data from a 2019 systematic review, the proportion of persons within CP cohorts reported to have a congenital anomaly ranged from 11.0% to 32.1%.^7^ The proportions varied between 1.5% and 18.0% for cerebral anomalies and between 2.1% and 18.9% for isolated non‐cerebral anomalies.^7^ Reported reasons for this wide variation included variability around inclusion criteria related to birth gestations, minor congenital anomalies, affected body systems, postneonatal insults, and CP severity. Studies also differed on whether they undertook additional linkage with a congenital anomaly register and, if so, the methodology used.^7^

To account for the methodological heterogeneity and to increase study power, a collaborative study was subsequently planned using a common methodology and linkage with the relevant congenital anomaly registers.^8^ Major congenital anomalies were present in 23% of the pooled data set for births in the years 1991 to 2009 from nine CP registers in Europe and Australia. Cerebral anomalies predominated; 14% had a cerebral anomaly and 10% an isolated cerebral anomaly.^6^ Of anomalies in non‐cerebral body systems, the most common were cardiac anomalies, present in 3.9% of the cohort. The presence of non‐cerebral anomalies raises questions about when the anomaly is on a causal pathway to CP (pathogenic) rather than being an alternative outcome of a common root cause or being causally unrelated to the development of CP (non‐pathogenic).

An earlier paper from Western Australia investigated potential reasons for an excess of non‐cerebral congenital anomalies in CP cohorts.^9^ A small proportion was thought to be due to ascertainment bias, as persons with CP were more likely to undergo detailed medical examination. The larger proportion was attributed to underdiagnosis of cerebral anomalies in persons with non‐cerebral defects. Further light may be shed on these uncertainties with the addition of confirmatory neuroimaging data. Magnetic resonance imaging data were largely unavailable at that time but are now reported for most persons with CP born since 1999 in the Australian state of Victoria. This has facilitated the development of a working classification of CP causal pathways and the creation of causal pathway subgroups to help elucidate how congenital anomalies might contribute to the development of CP.

Using population data from Victoria, we aimed to: (1) determine the proportion of persons with CP with a major congenital anomaly, overall, and by body system(s) affected; (2) compare risk factors for CP and clinical features of CP across groups stratified on the presence of a major congenital anomaly and multiple anomalies; and (3) identify the proportion of persons with anomalies that potentially contributed to the development of CP and the causal pathways involved.

## METHOD

### Setting and ethics

The setting for this population‐based, observational study was the Melbourne Children's campus in the Australian state of Victoria. Ethics approval for the Victorian Cerebral Palsy Register project was from The Royal Children's Hospital Human Research Ethics Committee. Consent was obtained for registration and use of register data but was not required specifically for this study.

### CP cohort

The CP cohort was identified from the Victorian Cerebral Palsy Register and included persons with CP born in Victoria between 1999 and 2017. The Victorian Cerebral Palsy Register project is an ongoing epidemiological study of persons with CP born in Victoria since 1970. Potential registrants are identified through active surveillance of medical records from relevant state‐wide paediatric services. After obtaining consent, data are collected for the Register from electronic medical records at the birth, neonatal, and paediatric hospitals, usually soon after diagnosis. The data are routinely updated from paediatric medical records when the child reaches 5 years of age. Reviewing of the medical records at ages 10, 15, and 20 years enables collection of missing data and new information from neuroimaging studies, genetic investigations, or procedures such as gastrostomy tube insertion.

### Data and definitions

Congenital anomalies were recorded in the Register database if reported in the person's birth, neonatal, or paediatric medical record. For comparability with recent research from Europe and Australia, included major congenital anomalies were based on criteria and definitions from the European Surveillance of Congenital Anomalies (EUROCAT).^10^ Each major congenital anomaly was classified as cerebral, cardiac, or other (non‐cerebral, non‐cardiac) according to the body system affected. Isolated patent ductus arteriosus was excluded. Cardiac anomalies were further subclassified as severe or non‐severe based on EUROCAT criteria. Severe anomalies include single ventricle, hypoplastic left or right heart syndrome, Epstein anomaly, tricuspid or pulmonary valve atresia, aortic valve/subaortic atresia or stenosis, common arterial truncus, atrioventricular septal defects, transposition of the great vessels, tetralogy of Fallot, total anomalous pulmonary venous return, and coarctation of the aorta/hypoplasia of the aortic arch. Each person was categorized as having no, single system, or multiple system anomalies.

In the Register data set, maternal age, parity, and plurality apply to the time of birth of the person with CP. Maternal grand multiparity was defined as five or more previous births at 20 or more gestational weeks. Parental consanguinity and dysmorphic features were recorded as present if described as such in the medical records. Condition at birth was deemed poor if any of the following criteria were met: 5‐minute Apgar score lower than 7, requirement for significant resuscitation (ventilation, continuous positive airway pressure, intubation, or chest compressions), or longer than 1 minute to establish regular respirations. Non‐spastic motor types include dyskinesia (dystonic and/or choreo‐athetotic subtypes), ataxia, and hypotonia. A quadriplegic motor type describes involvement of all four limbs. Gross Motor Function Classification System (GMFCS) levels III to V describe persons requiring mobility assistance, either with a walking aid or wheelchair. Epilepsy is defined as a history of two or more unprovoked seizures or a clinical diagnosis of epilepsy. A diagnosis of intellectual disability is based on an IQ below 70 on psychometric testing or on clinical grounds.

To explore some of the common causal pathways to CP, causal pathway subgroups were established based on predominant neuroimaging patterns classified according to the Magnetic Resonance Imaging Classification System.^11^ Causal pathway subgroups were broadly categorized as: (1) developmental (early prenatal structural or functional disruption to brain development); (2) late prenatal/perinatal destructive insults (white matter and grey matter injury patterns); and (3) brain insults that occurred postneonatally (after the 28th day of life). For more detailed analyses, predominant white matter injuries were further dichotomized based on birth gestation (<35, >35 weeks) and subcategories of predominant grey matter injuries were dichotomized as global hypoxic‐ischaemic insults and focal arterial infarctions.

Assessment of the potential for anomalies to have contributed to development of CP, or being pathogenic, was based on imaging evidence supporting the role of cerebral maldevelopments as a cause of CP,^12^ and on the potential for the anomaly, or the usual clinical management of the anomaly (often surgery), to be causally related to cerebral hypoxic‐ischaemic or haemorrhagic brain injury.

### Statistical analysis

All tabulations and statistical analyses were conducted using Stata 17.1 (Version 17; StatCorp, College Station, TX, USA). Odds ratios (OR) and the corresponding 95% confidence intervals (CI) were calculated to compare CP risk factors and clinical profiles across groups stratified on the presence/absence of a major congenital anomaly and across groups with one versus multiple anomalies. Records with missing data that were considered missing at random were removed from the analysis. Pathogenic anomalies were compared across body systems and causal pathway subgroups. A separate category was used for persons where the cause was unknown. A directed acyclic graph was constructed to show potential pathways involving congenital anomalies.^13^

## RESULTS

Of 2238 eligible persons with CP, 523 (23.4%) were recorded as having at least one major congenital anomaly based on EUROCAT criteria. Amongst these, cerebral anomalies were identified in 324 (14.5%) persons, cardiac anomalies in 122 (5.4%), and anomalies in other body systems in 153 (6.8%); 62 and seven persons respectively had major anomalies in two and three or more body systems.

Table 1 compares risk factors for CP and clinical features of CP between persons with and without at least one major congenital anomaly. The presence of a congenital anomaly was associated with higher frequencies of consanguinity (OR 2.4 [95% CI 1.4, 4.4]), maternal age under 20 years (OR 1.9 [95% CI 1.2, 3.1]), and maternal grand multiparity (OR 1.8 [95% CI 0.9, 3.5]). Clinically, persons with a major congenital anomaly were more likely to have dysmorphic features (OR 5.1 [95% CI 3.5, 7.4]), a non‐spastic motor type (OR 1.7 [95% CI 1.3, 2.2]), epilepsy (OR 2.1 [95% CI 1.7, 2.6]), bilateral (quadriplegic) CP (OR 2.0 [95% CI 1.6, 2.4]), intellectual disability (OR 3.1 [95% CI 2.4, 3.8]), and a GMFCS level between III and V (OR 1.8 [95% CI 1.5, 2.2]).

**TABLE 1:** Comparison of risk factors for CP and clinical features of CP across groups stratified on the presence/absence of a major CA and across groups with single system versus multisystem anomalies.

	No CA *n* = 1715	Any CA *n* = 523	Odds of any CA	Multisystem CAs *n* = 69	Single system CA *n* = 454	Odds of multisystem CAs
	*n* (col %)	*n* (col %)	OR (95% CI)	*n* (col %)	*n* (col %)	OR (95% CI)
Parental consanguinity	31 (2.2)	23 (5.2)	2.4 (1.4, 4.4)^a^	7 (10.1)	16 (3.5)	3.1 (1.0, 8.3)^a^
Maternal age < 20 years	53 (3.4)	30 (6.4)	1.9 (1.2, 3.1)^a^	3 (4.6)	27 (6.7)	0.6 (0.1, 2.2)
Maternal age > 35 years	392 (25.3)	112 (23.9)	0.9 (0.7, 1.2)	14 (21.2)	98 (24.4)	0.8 (0.4, 1.6)
Primiparity	696 (47.4)	186 (41.5)	0.8 (0.6, 1.0)^a^	23 (36.5)	163 (42.3)	0.8 (0.4, 1.4)
Grand multiparity	28 (1.9)	15 (3.3)	1.8 (0.9, 3.5)	3 (4.8)	12 (3.1)	1.6 (0.3, 6.0)
Multiple pregnancy	205 (12.0)	43 (8.2)	0.6 (0.4, 0.9)^a^	4 (5.8)	39 (8.6)	0.6 (0.2, 1.9)
Male sex	1007 (58.7)	292 (55.8)	0.9 (0.7, 1.1)	37 (53.6)	255 (56.2)	0.9 (0.5, 1.6)
Delivery < 35 weeks	625 (36.8)	123 (23.8)	0.5 (0.4, 0.7)^a^	16 (23.2)	107 (23.8)	1.0 (0.5, 1.8)
Poor condition at birth	793 (52.4)	158 (33.3)	0.5 (0.4, 0.6)^a^	30 (44.1)	128 (31.5)	1.7 (1.0, 3.0)^a^
NICU admission	761 (46.0)	175 (34.0)	0.6 (0.5, 0.7)^a^	32 (47.1)	143 (32.0)	1.9 (1.1, 3.3)^a^
Dysmorphic features	54 (3.2)	74 (14.2)	5.1 (3.5, 7.4)^a^	27 (39.1)	47 (10.4)	5.6 (3.0, 10.2)^a^
Non‐spastic motor type	232 (13.6)	110 (21.2)	1.7 (1.3, 2.2)^a^	21 (31.3)	89 (19.7)	1.8 (1.0, 3.4)^a^
Bilateral (quadriplegia) CP	542 (31.7)	249 (47.6)	2.0 (1.6, 2.4)^a^	46 (66.7)	203 (44.7)	2.5 (1.4, 4.4)^a^
GMFCS levels III–V	554 (32.5)	241 (46.4)	1.8 (1.5, 2.2)^a^	45 (66.2)	196 (43.4)	2.6 (1.4, 4.6)^a^
Epilepsy	519 (30.3)	249 (47.6)	2.1 (1.7, 2.6)^a^	37 (54.4)	212 (47.1)	1.3 (0.8, 2.3)
Intellectual disability	715 (45.6)	345 (71.9)	3.1 (2.4, 3.8)^a^	59 (92.2)	286 (68.8)	5.4 (2.1, 17.5)^a^

Abbreviations: CA, congenital anomaly; COL, column; CP, cerebral palsy; GMFCS, Gross Motor Function Classification System; NICU, neonatal intensive care unit; OR, odds ratio.

^a^ Denotes statistical significance at *p* ≤ 0.05.

### Congenital anomalies in single versus multiple body systems

A major congenital anomaly confined to a single body system was recorded for 454 (20.3%) persons whereas 69 (3.1%) had anomalies affecting multiple systems (Table 1). Multisystem anomalies were reported more frequently in association with parental consanguinity (OR 3.1 [95% CI 1.0, 8.3]), admission to neonatal intensive care (OR 1.9 [95% CI 1.1, 3.3]), dysmorphic features (OR 5.6 [95% CI 3.0, 10.2]), and CP of greater complexity, particularly concomitant intellectual disability (OR 5.4 [95% CI 2.1, 17.5]) but also bilateral (quadriplegic) CP, GMFCS levels III to V, and a non‐spastic motor type.

### Contribution of congenital anomalies to CP aetiology and causal pathways involved

#### Cerebral anomalies

All 324 (100%) persons with cerebral anomalies had anomalies that were deemed to be on a causal pathway to CP (Table 2). Developmental pathways were implicated for 306 (94.4%) persons, corresponding to 13.7% of the CP cohort. The 18 exceptions, involving different pathways, included 10 persons with congenital hydrocephalus after antenatal haemorrhage in late pregnancy, one with callosal dysgenesis but where neonatal kernicterus was considered causal, and seven with postneonatal insults. Of the latter group, three persons developed motor impairment after disconnection surgery for seizure disorders associated with malformations of cortical development and four persons had haemorrhagic strokes (three spontaneous; one surgical) associated with arteriovenous malformations.

**TABLE 2:** Number and proportions of persons with pathogenic congenital anomalies by body system and across causal pathway subgroups of CP.

Causal pathway subgroup	Pathogenic CA: cerebral	Pathogenic CA: cardiac	Pathogenic CA: other system CA	Pathogenic CA: any body system
	*n* (%)	*n* (%)	*n* (%)	*n* (%)
Developmental	306 (94.4)	0 (0.0)	0 (0.0)	306 (81.6)
Insults <35 weeks	0 (0.0)	6 (14.0)	4 (50.0)	10 (2.7)
PWMI	0 (0.0)	4 (9.3)	3 (37.5)	7 (1.9)
Other	0 (0.0)	2 (4.7)	1 (12.5)	3 (0.8)
Insults >35 weeks	11 (3.4)	17 (39.5)	2 (25.0)	30 (8.0)
PWMI	0 (0)	5 (11.6)	0 (0.0)	5 (1.3)
HIE	0 (0)	8 (18.6)	1 (12.5)	9 (2.4)
AIS	0 (0)	4 (9.3)	1 (12.5)	5 (1.3)
Other	11 (3.4)	0 (0.0)	0 (0.0)	11 (2.9)
Postneonatal insults	7 (2.2)	20 (46.5)	1 (12.5)	28 (7.5)
Other/unknown	0 (0)	0 (0.0)	1 (12.5)	1 (0.2)
All subgroups	324 (100)	43 (100)	8 (100)	375 (100)
% of CP cohort	14.5%	1.9%	0.4%	16.8%

Abbreviations: AIS, arterial ischaemic stroke; CA, congenital anomaly; CP, cerebral palsy; HIE, hypoxic‐ischaemic encephalopathy; PWMI, predominant white matter injury.

#### Cardiac anomalies

Overall, 43 persons had cardiac anomalies deemed potentially contributory to CP aetiology (pathogenic), corresponding to 35.2% of those with cardiac anomalies and 1.9% of the CP cohort (Table 2). Only four of these persons had non‐severe cardiac anomalies, these being associated with postneonatal insults. The most common severe cardiac anomalies were transposition of the great vessels (*n* = 8), coarctation of the aorta (*n* = 9), hypoplastic left heart syndrome (*n* = 7), and tetralogy of Fallot (*n* = 6). Potentially pathogenic cardiac anomalies were predominantly associated with either postneonatal insults or pre/perinatal destructive insults in persons born near term. Postneonatal insults included 14 ischaemic strokes (two perioperative), five hypoxic‐ischaemic encephalopathic events, and one cardiac arrest.

#### Other anomalies

Of the 153 persons with anomalies in other body systems, 39 also had cerebral anomalies and 24 had additional cardiac anomalies; seven had both cerebral and cardiac anomalies (Table 2). Only 5.2% of persons with non‐cerebral/non‐cardiac anomalies and 0.4% of the whole cohort had anomalies that were thought to contribute to CP aetiology. The pathogenic anomalies included craniosynostosis, oesophageal atresia with fistula, and gastroschisis.

**FIGURE 1:**
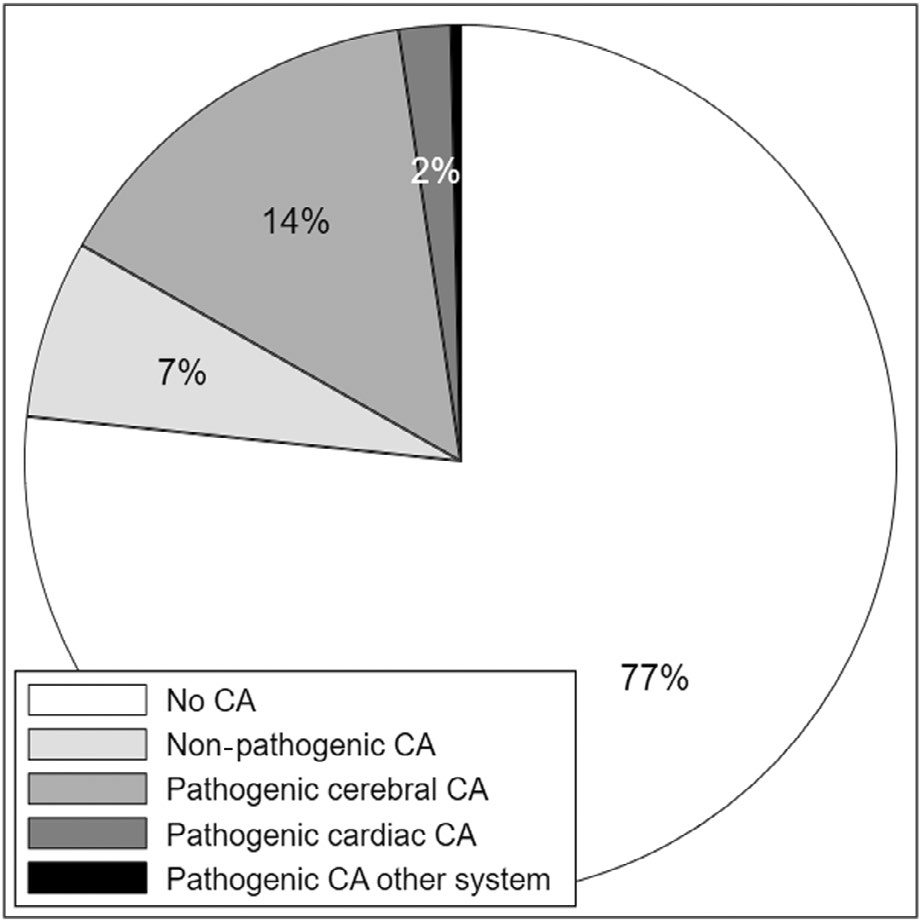
Proportions of persons with cerebral palsy with no, non‐pathogenic and potentially pathogenic cerebral, cardiac, and other body system congenital anomalies (CA).

**FIGURE 2:**
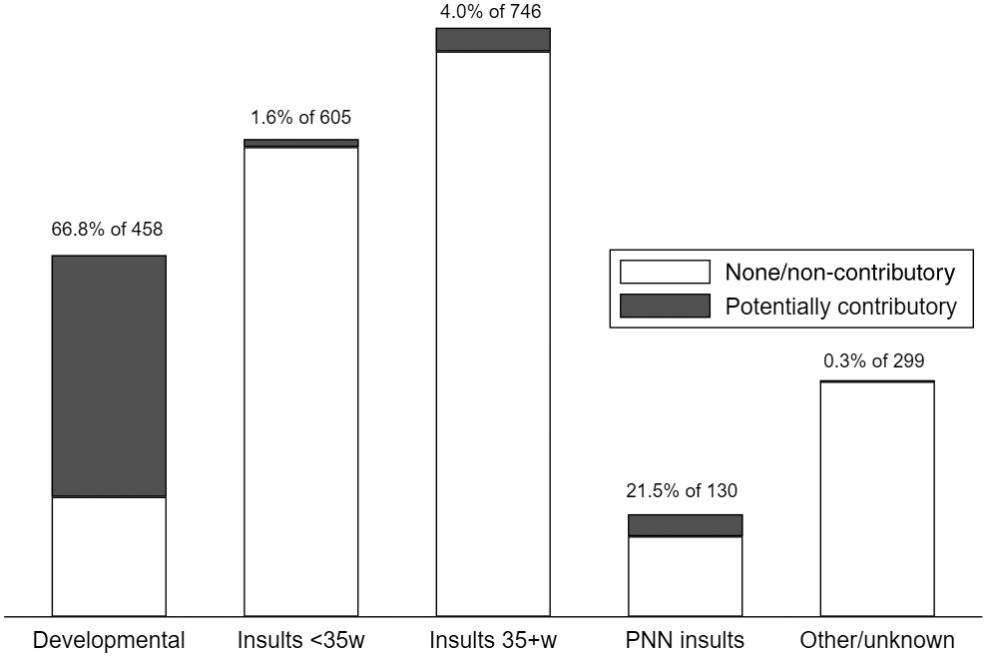
Distribution of persons within a cerebral palsy cohort by causal pathway subgroup, and proportion of each subgroup with pathogenic anomalies.

**FIGURE 3:**
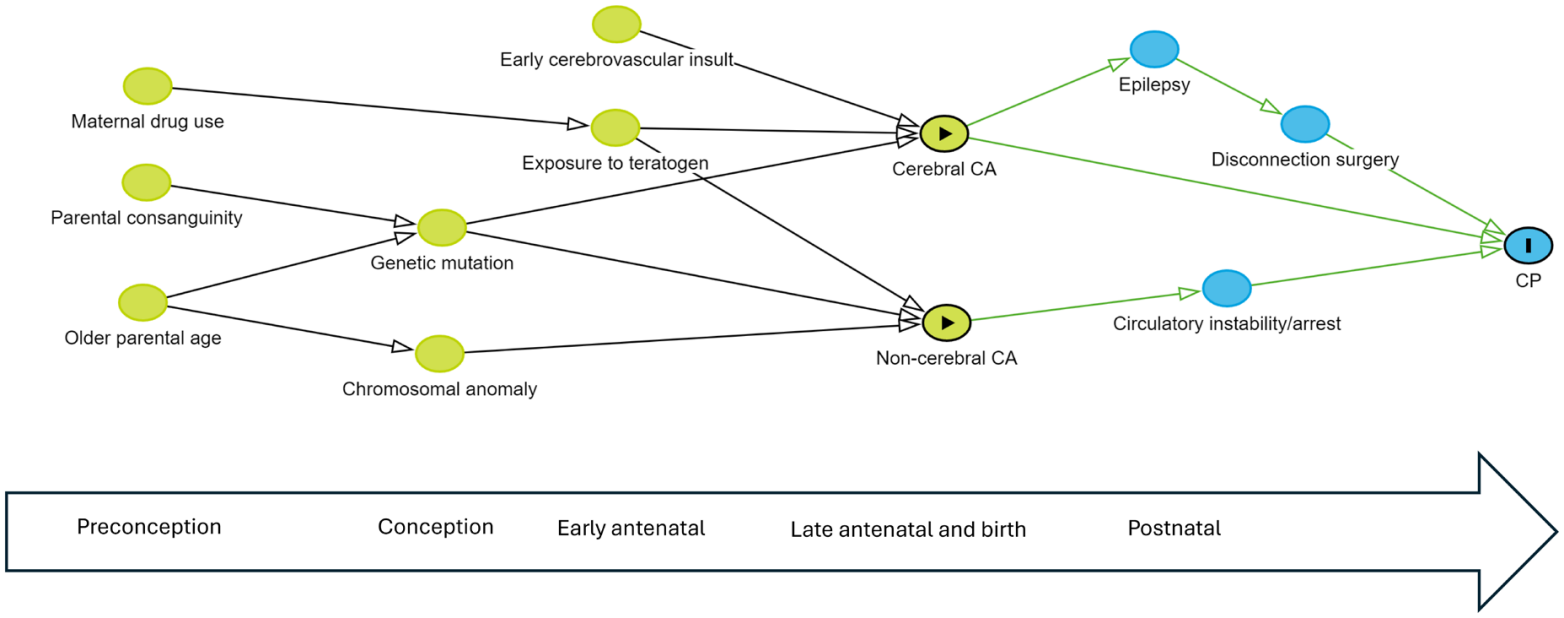
Directed acyclic graph showing potential pathways to cerebral palsy (CP) involving major congenital anomalies (CA).

In summary, 76.6% of the CP cohort did not have a major congenital anomaly, 6.6% had a non‐pathogenic anomaly, and 16.8% had a potentially pathogenic anomaly (Figure 1). Anomalies in the latter group comprised 14.5% cerebral, 1.9% cardiac, and 0.4% other body system (Table 2). Of the 14.5% with cerebral anomalies, 13.7% contributed to CP aetiology through developmental pathways and 0.8% through other causal pathways. Conversely, 66.8% of persons with developmental anomalies and 21.5% of those with postneonatal insults had congenital anomalies that were likely contributory to CP aetiology (Figure 2). The proportions were small in the other causal pathway subgroups. Potential causal pathways involving major congenital anomalies are depicted in the directed acyclic graph (Figure 3).

## DISCUSSION

In our large, population‐based CP cohort, 23% of persons had a major congenital anomaly and 17% had a major anomaly that potentially contributed to the development of CP. The majority (82%) of pathogenic anomalies, present in 14% of the cohort, involved developmental causal pathways and cerebral anomalies. Less than 3% had anomalies that were on causal pathways to CP involving late prenatal, perinatal, or postneonatal destructive brain insults. Most of these were severe cardiac anomalies.

The study has several limitations. There were varying amounts of missing data that may have affected precision around the calculated odds ratios if missing data were not entirely missing at random. There was also the potential for under‐reporting of major anomalies that did not require diagnosis or intervention from tertiary health institutions. We believe the magnitude of under‐reporting would be small as the proportion of major anomalies in another Victorian CP cohort was previously assessed through linkage with the state's Congenital Anomalies Register and was found to be comparable to the findings of this study.

Our study used the same EUROCAT eligibility criteria for major congenital anomalies as was used in a recent European and Australian data linkage study which also included data from Victoria.^6^ Both studies arrived at the same percentage of persons with major anomalies, although we also included 7% of persons with brain insults acquired postneonatally. The proportion of CP with cerebral anomalies was 13.9% in the pooled data set and 14.5% in this study. In both studies, cardiac anomalies were the most common non‐cerebral anomalies, occurring in 3.9% of the pooled data set and in 5.4% of ours. This latter difference is likely partly explained by the fact that a proportion of persons with severe cardiac anomalies in the earlier study were classified as having a chromosomal, genetic, or teratogenic syndrome and were, thereby, not included in the subgroup with cardiac anomalies, the categories being mutually exclusive. Another potential explanation for the higher frequency in our Victorian cohort is in utero transfer from other Australian states of fetuses with severe cardiac anomalies for delivery and postnatal management.

Both the data linkage study^6^ and this study found that anomalies were more common in singleton and term/late‐term births, with no meaningful sex predominance. Both studies also showed that persons with a congenital anomaly were more likely to have a complex CP phenotype. In addition, we reported associations between the presence of a congenital anomaly and consanguineous parentage, maternal age under 20 years, maternal grand multiparity, and dysmorphic features. Consanguinity, admission to neonatal intensive care, dysmorphic features, and complex CP were relatively more frequent in the subgroup with multisystem anomalies. Congenital anomalies have been previously associated with consanguinity, older maternal age, and grand multiparity,^14^, ^15^ but not with adolescent pregnancy.

Our classification of causal pathway subgroups includes developmental causes of CP in approximately 20% of the CP cohort. Imaging studies show that around 10% to 15% of CP is due to brain maldevelopment, mostly within the spectrum of malformations of cortical development.^11^, ^12^, ^16^ Reasons for the 5% to 10% difference include missed abnormalities because of their subtlety or suboptimal timing of diagnostic imaging,^17^ or to deficits that are functional rather than structural, as is often the case with chromosomal/genetic syndromes. This explains why there is a cerebral anomaly in only two‐thirds of persons with developmental causes of CP, and the fact that 30% to 40% of non‐cerebral anomalies were in persons with developmental causes of CP, despite not being directly contributory to its aetiology.

Birth before 35 weeks' gestation increases the risk of brain insults, particularly white matter injury. The congenital anomalies in this causal pathway subgroup were all non‐cerebral, with most cardiac anomalies being non‐severe, isolated septal defects, or pulmonary stenosis.^18^ This finding can be partly explained by the fact that infants born preterm are likely to be subject to intense diagnostic investigations, identified anomalies often being of little clinical significance. Consequently, whereas 11% of this subgroup had a congenital anomaly, only 15% were deemed likely pathogenic, corresponding to only 0.4% of CP. The congenital anomalies that were potentially pathogenic may have directly affected the pregnancy. For example, congenital heart disease has been associated with polyhydramnios,^19^ preeclampsia,^20^ and smaller placental, fetal, and brain size.^21^ These may, in turn, increase the risk of fetal brain injury or preterm delivery. Fetuses with specific non‐cerebral anomalies have been shown to have a higher risk of preterm delivery,^22^ with the association being strongest for spontaneous rather than indicated deliveries.^23^, ^24^ Severe defects, a more complicated neonatal period, and need for clinical management of the congenital anomaly are likely to amplify the existing risk of white matter injury in neonates born preterm, particularly in those requiring surgery.^25^, ^26^

In this study, only a small proportion of CP (0.3%) was associated with pathogenic congenital anomalies in the causal pathway subgroups of CP associated with birth nearer term and with a generalized or focal cerebral insult acquired prenatally or perinatally. Some persons had neuroimaging evidence of white matter injury that may reflect chronic or milder degrees of cerebral hypoxia/ischaemia occurring before surgery to correct the anomaly.^27^ Non‐cerebral congenital anomalies have also been shown to be the likely cause of neurodevelopmental sequelae subsequent to neonatal encephalopathy,^28^ and cardiac anomalies have been associated with perinatal ischaemic stroke.^29^

Postneonatal events comprise only about 6% of all CP in developed countries, but approximately one‐quarter of persons classified to this causal pathway subgroup had a congenital anomaly in both the present study and the data linkage study.^30^ Cardiac anomalies predominated in both studies, with interventions frequently implicated. Currently, classifications of postneonatal causes of CP used by many CP registers does not enable an accurate estimate of the contribution of congenital anomalies.^31^ Resolving this limitation in future versions would facilitate cross‐referencing.

Recent research has unmasked potential reasons for variations in reports of the frequency of congenital anomalies in CP and standardized methodologies have been established. Consequently, there is now good evidence that major congenital anomalies are present in nearly a quarter of persons with CP. Through neuroimaging studies, it is also known that cerebral anomalies are associated with 10% to 15% cases of CP. The question that remained to be answered was to what extent, and via what causal pathways, do other congenital anomalies contribute to CP aetiology? This study takes that next step forward by undertaking a deeper dive into the contribution of congenital anomalies to causal pathways in CP in a large population cohort. The population perspective provides context for the impact on CP rates of preventive measures or other changes in incidence or management of congenital anomalies.

## CONFLICT OF INTEREST STATEMENT

All authors declare that they have no conflicts of interest.

## Data Availability

The data that support the findings of this study are available from the corresponding author upon reasonable request.
